# Supplementary material for: DNA-Binding Properties of a Novel Crenarchaeal Chromatin-Organizing Protein in Sulfolobus acidocaldarius
Source: Biomolecules. 2022 Mar 30;12(4):524. doi: 10.3390/biom12040524 (PMC9025068; doi:10.3390/biom12040524)
Supplement: Supplementary file 1 [file biomolecules-12-00524-s001.zip › biomolecules-1601255-supplementary.pdf]

biomolecules

Supplementary Materials

Table S1. Overview of oligonucleotides used in this work.

Name	Sequence (5′ to 3′)	Purpose
LL171	TTATGCATTGGAAAGAGACGA	EMSA Target *Saci_1374*
LL172	CCCTTAATCTCCTTATTGCTTCG	EMSA Target *Saci_1374*
LL173	CCCTCCATTTGGAGTAGTGAA	EMSA Target *Saci_0017*
LL174	AGTGTTGCAAATGCCCTACC	EMSA Target *Saci_0017*
LL175	GCCTTTTCGATCGTATCATCA	EMSA Target *Saci_1506*
LL176	TTGCGTTTTCAAGACCAGAA	EMSA Target *Saci_1506*
LL177	TCAAAGCTGATCCCCTCACT	EMSA Target *Saci_1045*
LL178	CTTCACCCCTGAGGCTTATG	EMSA Target *Saci_1045*
LL179	CTGGCATTGGCAAAGGTTAT	EMSA Target *Saci_2344*
LL180	GGATGCTGAGGTCCAACATT	EMSA Target *Saci_2344*
LL181	TGCAGACGAGGATTTGATGA	EMSA Target *Saci_2246*
LL182	ATAGCCCATGCAGGGAGAC	EMSA Target *Saci_2246*
LL183	ATGTCCGTTTTGCCTTTTTG	EMSA Target *Saci_1906*
LL184	CGACAACAACGATCTCATTCA	EMSA Target *Saci_1906*
LL185	CATGGAGGACTCAAGCAGGT	EMSA Target *Saci_1947*
LL186	TCTCCTCTGTCTCCCTCAAGA	EMSA Target *Saci_1947*
LL187	TGTACCTCGAAGAGGGGAAA	EMSA Target *Saci_1872*
LL188	CTTCCTCTTCCGGAATACCC	EMSA Target *Saci_1872*
LL189	AAACCATTCCAGCCATTCAA	EMSA Target *Saci_0991*
LL190	TCCCTTCAAGAGGGAATCTG	EMSA Target *Saci_0991*
LL191	GGAGCATGGGATAGTGGAGA	EMSA Target *Saci_1353*
LL192	GCTAATCCCATGACGCCTAA	EMSA Target *Saci_1353*
LL193	CAACACGTAAAGGATTCCTCGT	EMSA Target *Saci_0472*
LL194	CTTGCTGAGGTGCAGATGAA	EMSA Target *Saci_0472*
LL195	TAGAATAAGAGATTATTGGGTCTTGTC	EMSA Target *Saci_1237*
LL196	CACAGCTGTAATAGGTTCCTTC	EMSA Target *Saci_1237*
LL197	TCCGGAAGAAGGTGAAAAGA	EMSA Target *Saci_1041*
LL198	TTCCGGCAATTATTGAGACC	EMSA Target *Saci_1041*
LL199	TGTCGCTTTTGACATTCACAGA	EMSA Target *Saci_1339*
LL200	AACCGACCAGCTTCAACAAG	EMSA Target *Saci_1339*
LL140	GATGGGAGTCTTGGACTCTAGG	EMSA Control *Saci_1851*
LL154	CTTACCTTTTATAGTAGGTAAATAAGG	EMSA Control *Saci_1851*

(a)

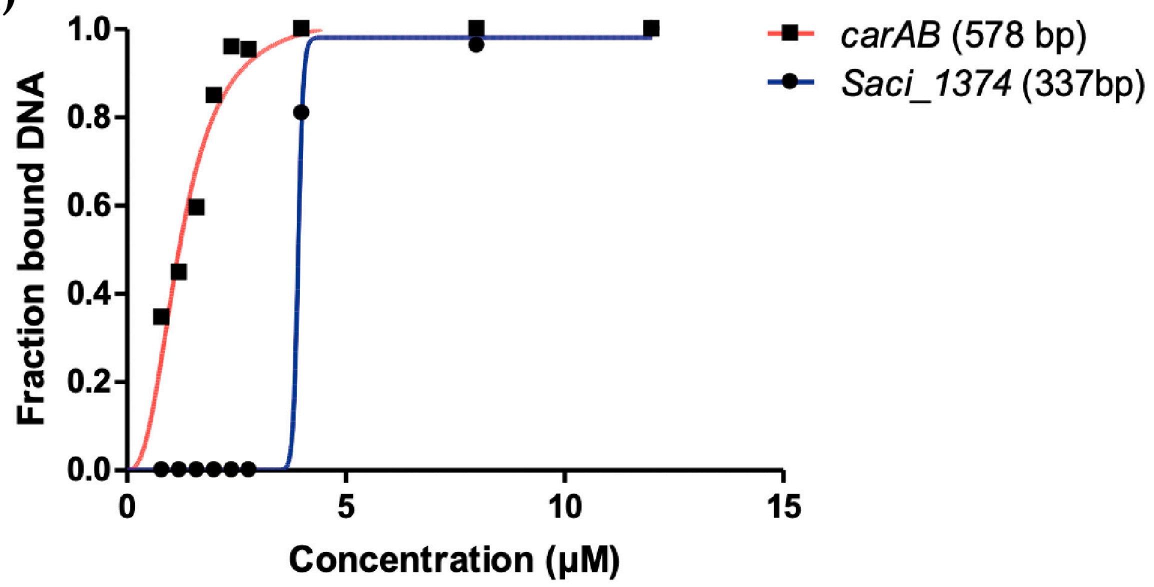

(b)

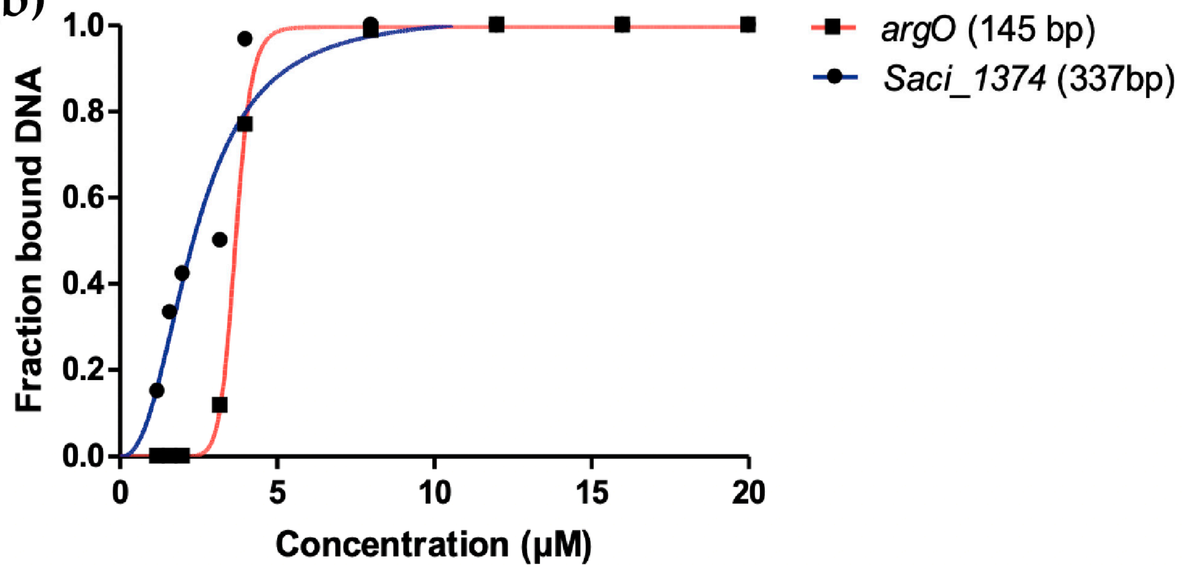

Figure S1. Fitted binding curves of EMSAs performed to study competition in Sul12a binding for different DNA probes with panel (**a**) corresponding to the EMSA in **Figure 6a** and panel (**b**) corresponding to the EMSA in **Figure 6b**. Calculated K_{Dapp}s are displayed in **Table 2**.
